# Spatiotemporal Pattern Evolution of Food and Nutrient Production in China

**DOI:** 10.3390/foods12203791

**Published:** 2023-10-16

**Authors:** Yumei Qi, Wenli Qiang, Xing Ma

**Affiliations:** College of Earth and Environmental Sciences, Lanzhou University, Lanzhou 730000, China; qiym21@lzu.edu.cn (Y.Q.); 220220949091@lzu.edu.cn (X.M.)

**Keywords:** food, nutrient, gravity center, food security, China

## Abstract

Ensuring food and nutrient supply is a crucial aspect of achieving food safety. With rapid population growth, urbanization, and social and economic development, the challenges related to China’s food and nutrient production have become increasingly prominent. This paper analyzed the characteristics of the spatiotemporal pattern evolution of food and nutrient production in China from 1995 to 2020, utilizing the conversion of various food nutrients and the establishment of a gravity center model. The results showed that: (1) Food production exhibited increasing trends in China, six regions, and 90% of the provinces. Notably, the structure of food production underwent significant changes in China, East China, Central-South China, Southwest China, Northwest China, and 60% of the provinces. (2) The output of all categories of food nutrients demonstrated increasing trends in China, six regions, and most provinces. At three different geographical scales, the changes of six food nutrients production structure showed significant differences. (3) Natural, political, social, economic, and technological factors played pivotal roles in influencing the gravity centers of food and nutrient production in China. The gravity centers of cereal production shifted northeast, while those of root and tuber, oil crops, and stimulants production moved westward. Additionally, the gravity centers of sugar crop, pulse, vegetable, fruit, and aquatic product production moved southwest and those of livestock and poultry production shifted northward. (4) Affected by the food production, the gravity centers of food energy, food protein, and food carbohydrate production shifted northward, while those of food fat, food vitamins, and food minerals production shifted northwest, southwest, and westward, respectively. The results of this study are of great significance for policy adjustments pertaining to the distribution pattern of food production, food security stability, and sustainable development in China.

## 1. Introduction

As defined by the World Food Summit, food security exists when all people at all times have access to adequate levels of safe, nutritious food for an active and healthy life [1]. This definition emphasizes the importance of both food and food nutrition. First, the rapid growth of the Chinese population since the reform and opening up has led to increased demands for food production. According to projections by the United Nations, China’s population is estimated to reach 1.44 billion by 2030 [2], with a peak expected between 2025 and 2043, ranging from 1.4 to 1.5 billion [3,4]. Second, China’s rapid urbanization has profound impacts on the food supply. The United Nations predicts that China’s urbanization rate will reach 69% by 2030 and will continue to rise thereafter [5]. This will further influence China’s agricultural planting layout [6], consumption structure [7], and ultimately, indirectly impact food production. Third, with the progress of society and economy, Chinese residents are increasingly prioritizing their physical health, leading to significant changes in dietary structure [8,9]. The issue of nutrition has become a focal point of attention [10].

Numerous studies have examined the changes in food production output in China, with a particular focus on grain production. For instance, Chen et al., have derived the conclusion of an increase in China’s overall grain production from 1980 to 2007 through the calculation of various indices, including the multiple cropping index [11]. Similarly, Pan et al. found that grain production exhibited an upward trend from 2000 to 2014 [12]. Li et al. conducted a study indicating that grain production displayed an overall upward trend from 2001 to 2019, but it experienced four stages: rapid decline, rapid growth, steady growth, and slow decline [13]. Additionally, Li et al., reported a five-fold increase in total grain output from 113 million tons in 1949 to 571 million tons in 2011 [14]. With regard to the quantitative variation of other food production, various scholars have observed distinct patterns. Vegetable production demonstrated increasing trends from 1990 to 2010 [15]. Apple production, on the whole, exhibited fluctuating growth after 1978 [16]. Tea production increased from 0.683 million tons in 2000 to 2.249 million tons in 2015, with an average annual growth rate of 8.3% [17]. Pork, beef, and mutton production were on the rise, while their changing rates were discrepant in different periods [18]. Furthermore, a considerable upward trend in total production of aquatic products was observed after 1998 [19].

Studies examining the spatial pattern evolution of food production in China have yielded varying conclusions. In terms of grain production, some studies have indicated an overall northward shift in gravity centers [20,21]. Additionally, scholars have conducted research on specific grain groups, revealing that the concentration ratio and scale index of corn production increased in the north, while decreasing in the south [22]. Regarding other food production, researchers have drawn the following conclusions: after 1978, sugarcane production gradually concentrated in Guangxi and Yunnan, and sugar beet production concentrated in Xinjiang and Inner Mongolia [23]; vegetable production increasingly concentrated in the Huang-Huai-Hai area and the Yangtze River Basin [24]; the distribution center of apple production shifted southwest [16]; tea production shifted from the eastern region to the central and western regions, expanding northward in terms of spatial distribution [6]; the gravity centers of pork, beef, and mutton production shifted southeast, west and north in Hubei, Shanxi, and Shaanxi, respectively [18].

In studies regarding the characteristics of the spatiotemporal pattern evolution of food nutrient production in China, Wang et al. concluded that the gravity centers of food energy, food protein, and food fat shifted northward, and the gravity centers of population shifted southward in China [20]. Zhang et al. investigated China’s calorie production in relation to grain, oil, sugar, fruit, and vegetable production, revealing a linear growth trend [25].

Other studies have also explored food production in different regions. Global grain production has exhibited a steady increase from 1992 to 2020. However, the movement of its gravity center is complicated [26]. Moreover, the annual average maize and soybean yields in the United States have shown a consistent upward trend from 1958 to 2007. Specifically, corn yields in the midwestern states of the United States have shown faster growth compared to the marginal southeastern regions. A similar spatial pattern can be observed in soybean production as well [27]. In the Matopiba region of Brazil, soybean production experienced substantial growth from 1990 to 2015. The production and yield during this period exhibited spatial patterns corresponding to local or regional aptitudes [28]. With regard to the spatiotemporal trends in the adequacy of dietary nutrient production and food sources, an assessment was conducted on the nutrient adequacy of primary production for 177 countries from 1995 to 2015. The findings indicate a consistent and steady increase in global nutrient production over the past two decades. Depending on changes in the food production basket, specific trends in nutrients varied by region [29].

However, most existing studies have primarily focused on the spatiotemporal pattern evolution of production of a single food group. As a result, the scope of the involved food groups has been limited, and considerations for food nutrient production, particularly vitamins and minerals, have been lacking. Therefore, building upon existing research, this article analyzed the characteristics of the spatiotemporal pattern evolution of food and nutrient production in China, by converting various food nutrients and constructing a gravity center model. This study intends to comprehensively consider issues related to food and nutrient production and provide valuable insights for policymaking regarding the distribution pattern of food production, ensuring food safety stability, and promoting sustainable development in China.

## 2. Materials and Methods

### 2.1. Materials

Referring to the food classification of the Food and Agriculture Organization of the United Nations, this study categorized food into 10 groups: cereals, roots and tubers, sugar crops, pulses, oil crops, vegetables, fruit, stimulant crops, livestock and poultry, and aquatic products, including 43 food items in total (Table 1).

Food production data were obtained from the National Bureau of Statistics (https://data.stats.gov.cn) (accessed on 9 September 2022). The conversion factors for energy, protein, carbohydrate, fat, vitamins, and minerals hidden in various food items were derived from the China Food Composition Tables (6th edition) [30] and the Food and Nutrient Database for Dietary Studies from the United States Department of Agriculture (https://www.ars.usda.gov) (accessed on 20 September 2022) [31]. Among them, vitamins include vitamin A, B, C, D, E, and K. Vitamin B involved in this paper includes vitamin B1 (thiamine), vitamin B2 (riboflavin), vitamin B3 (niacin), vitamin B6, vitamin B9 (folate), and vitamin B12. Vitamin B5 (pantothenic acid) and vitamin B7 (biotin) are not involved. Minerals include calcium, phosphorus, magnesium, iron, zinc, copper, selenium, potassium, sodium, and manganese. This paper divided 31 provinces (municipalities and autonomous regions) into six regions according to the National Bureau of Statistics (Table 2).

### 2.2. Methods

In physical terms, the center of gravity refers to the point at which the sum of the weighted relative positions of the distribution gravity equals zero [32]. The gravity center model is widely used in spatial analysis problems, such as population [20,33], urban land [34], tourist attractions [35], facility location [36], CO_2_ emission [37], and energy production and consumption [38], and it is an important analytical tool to study the spatial changes of factors in the process of regional development [39]. This paper constructed the gravity center model of food and nutrient production in China during 1995–2020:

(1) $$X_{j}\left(t\right)=\frac{\sum_{i=1}^{n}\left[P_{ij}\left(t\right)\cdot X_{i}\right]}{P_{j}\left(t\right)}$$

(2) $$Y_{j}\left(t\right)=\frac{\sum_{i=1}^{n}\left[P_{ij}\left(t\right)\cdot Y_{i}\right]}{P_{j}\left(t\right)}$$

where X_j_(t) and Y_j_(t) represent the abscissa and ordinate of food group or nutrient type j in year t, respectively. P_ij_(t) denotes the production of food group or nutrient type j in province i in year t. X_i_ and Y_i_ represent the abscissa and ordinate of the center of province i, respectively. P_j_(t) means the national total production of food group or nutrient type j in year t. n is the number of provinces.

Assuming that the coordinates of the gravity center of a certain food or nutrient production in China in year t and year t + m are C_t_ (X_t_, Y_t_) and C_t + m_ (X_t + m_, Y_t + m_), respectively, then the angle α and distance D of the gravity center C_t_ moving to the gravity center C_t + m_ from year t to year t + m can be calculated by Formula (3) and (4), respectively:

(3) $$\alpha \left(m\right)=\arctan\left(\frac{Y_{t+m}-Y_{t}}{X_{t+m}-X_{t}}\right)$$

(4) $$D\left(m\right)=\sqrt{{\left(X_{t+m}-X_{t}\right)}^{2}+{\left(Y_{t+m}-Y_{t}\right)}^{2}}$$

## 3. Results

### 3.1. Characteristics of Changes in Food Production

**Figure 1:**
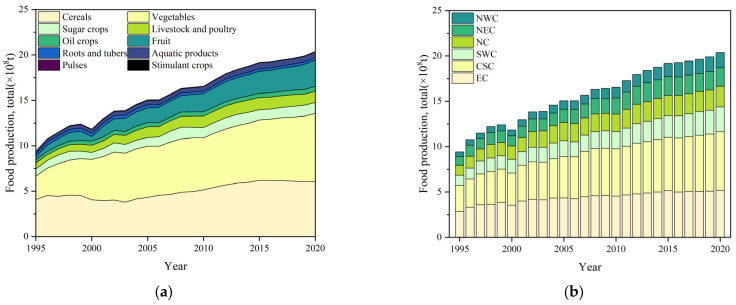
Changes of food production in China by (**a**) food group and (**b**) region from 1995 to 2020.

National food production showed a significant upward trend from 1995 to 2020 (Figure 1a), increasing from 9.41 × 10^8^ tons in 1995 to 20.38 × 10^8^ tons in 2020, with an increase of 1.17 times. Fruit and stimulant crop production increased by 7.36 times and 3.98 times, respectively. It was followed by vegetable and aquatic products, which increased by 1.91 and 1.57 times. With a smaller growth rate, livestock and poultry, oil crop, sugar crop, and cereal production increased by 0.87, 0.54, 0.51, and 0.49 times, respectively. In contrast, pulse and root and tuber production decreased slightly, by 25.16% and 8.44%, respectively.

From 1995 to 2020, the national food production structure changed significantly. In 1995, cereal and vegetable production accounted for 43.28% and 27.34% of the national total food production, respectively. Sugar crop, livestock and poultry, oil crop, fruit, root and tuber, aquatic product, pulse, and stimulant crop production accounted for 8.44%, 6.95%, 3.83%, 3.65%, 3.47%, 2.52%, 0.46%, and 0.06%, respectively. However, in 2020, vegetable, cereal, and fruit production accounted for 36.76%, 29.77%, and 14.08% of the national total food production, respectively. Livestock and poultry, sugar crop, aquatic product, oil crop, root and tuber, pulse, and stimulant crop production accounted for 6.00%, 5.90%, 3.00%, 2.72%, 1.47%, 0.16%, and 0.14%, respectively. The proportion of cereal, root and tuber, sugar crop, pulse, oil crop, and livestock and poultry production decreased, and the proportion of cereal production had the largest decrease of 13.50%. The proportion of fruit and vegetable production increased significantly, by 10.43% and 9.42%, respectively.

Food production in all regions showed increasing trends (Figure 1b). From 1995 to 2020, the food production in NWC increased by 2.49 times. It was followed by that in SWC, CSC, NC, and NEC, which increased by 1.44, 1.27, 1.08, and 1.04 times, respectively. With a smaller growth rate, that in EC increased by 0.81 times. In NWC, the food production increase mainly came from vegetable and fruit production increases, which accounted for 39.58% and 35.46% of the food production increase, respectively. In SWC, the food production increase mainly came from the vegetable production increase, accounting for 63.61%, followed by the fruit production increase, accounting for 18.57%. In CSC, the food production increase mainly came from the vegetable and fruit production increases, accounting for 45.36% and 25.06%, respectively. In NC, the food production increase mainly came from the vegetable and cereal production increases, accounting for 31.51% and 29.52%, respectively. In NEC, the food production increase mainly came from cereal production increase, accounting for 69.73%. In EC, the vegetable production increase accounted for more than half of the food production increase, followed by the fruit production increase, accounting for 24.98%.

**Figure 2:**
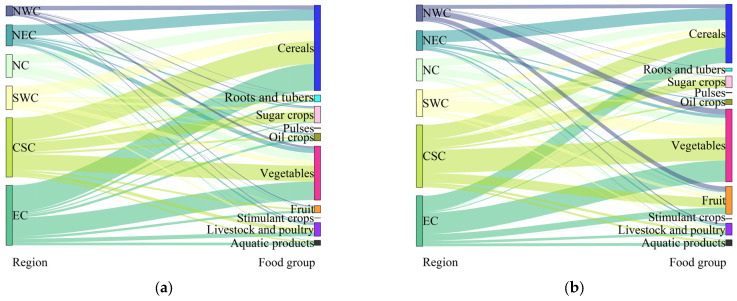
Food production structure of the six regions in (**a**) 1995 and (**b**) 2020.

The changes of the food production structure in the six regions showed significant differences (Figure 2). From 1995 to 2020, the proportion of cereal production in NC was always the highest, followed by that of vegetable production, but the proportion of cereal and vegetable production decreased from 42.01% and 35.19% to 35.52% and 33.28%, respectively. The cereal production in NEC always accounted for more than half of NEC’s total food production and increased from 52.16% in 1995 to 61.10% in 2020. In EC, CSC, and SWC, the food group with the highest proportion of production changed from cereal to vegetable, and the proportion of cereal production decreased from 44.16%, 38.38%, and 46.67% in 1995 to 30.58%, 22.71%, and 20.91% in 2020, respectively. In contrast, the proportion of vegetable production increased from 31.04%, 26.80%, and 19.15% to 42.32%, 37.19%, and 45.35%, respectively. In 1995, cereal was the food group with the highest proportion of production in NWC, followed by vegetable, accounting for 43.30% and 22.42%, respectively. However, in 2020, the food group with the highest proportion of production in NWC changed to vegetable, followed by fruit and cereal, accounting for 34.67%, 27.86%, and 23.23%, respectively.

During the study period, food production in 90% of the provinces showed increasing trends. The food production of Xinjiang, Ningxia, Inner Mongolia, Shaanxi, Gansu, Guizhou, and Henan increased by more than twice. In Inner Mongolia, the food production increase mainly came from the cereal production increase, accounting for 46.19%. In Guizhou, Henan, Ningxia, and Gansu, the food production increase mainly came from the vegetable production increase, accounting for 75.88%, 46.70%, 45.65%, and 44.80%, respectively. In Shaanxi and Xinjiang, the fruit production increase was the main source of food production increase, accounting for 45.64% and 36.09%, respectively, followed by the vegetable production increase, accounting for 40.41% and 33.10%, respectively.

The changes of the food production structure also showed significant differences among provinces. Cereal was always the food group with the highest proportion of production in Jilin, Heilongjiang, Anhui, Jiangxi, Inner Mongolia, Shanxi, and Liaoning from 1995 to 2020. The proportion of cereal production in Jilin and Heilongjiang increased from 66.24% and 53.43% in 1995 to 76.20% and 70.65% in 2020, respectively. In contrast, it decreased from 54.59%, 49.06%, 48.20%, 46.20%, and 39.14% to 49.08%, 39.64%, 46.75%, 35.76%, and 35.59% in Anhui, Jiangxi, Inner Mongolia, Shanxi, and Liaoning, respectively. In Guangxi, the proportion of sugar crop production was always the highest and close to 45%. In Shanghai, Guangdong, Tianjin, and Beijing, vegetable was always the food group with the highest proportion of production. The proportion of vegetable production in Shanghai and Guangdong increased from 43.64% and 28.50% in 1995 to 55.83% and 39.16% in 2020, respectively. On the contrary, it decreased from 59.89% and 51.47% to 40.01% and 49.04% in Tianjin and Beijing, respectively. In addition, the food group with the highest proportion of production changed from cereal to vegetable in 50% of the provinces. In Hainan and Yunnan, the food group with the highest proportion of production changed from sugar crop to vegetable. The proportion of sugar crop production decreased from 43.76% and 37.48% in 1995 to 7.01% and 21.17% in 2020, while that of vegetable production increased from 16.90% and 14.39% to 37.96% and 33.24%, respectively. In 1995, the proportion of cereal production in Shaanxi was the highest (45.99%), but fruit became the food group with the highest proportion of production in 2020, followed by vegetable, accounting for 36.83% and 34.83% respectively.

### 3.2. Characteristics of Changes in Nutrient Production

**Figure 3:**
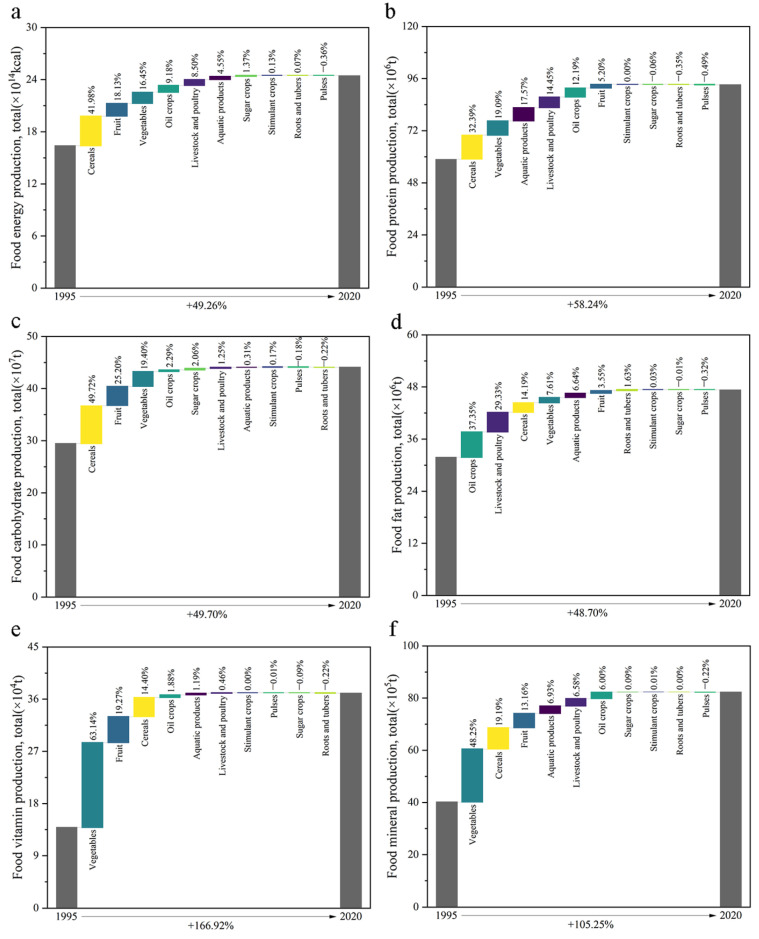
Changes of (**a**) food energy, (**b**) food protein, (**c**) food carbohydrate, (**d**) food fat, (**e**) food vitamin, and (**f**) food mineral production in China from 1995 to 2020.

At the national scale, the production of all types of food nutrients showed increasing trends (Figure 3). Among them, the production of food vitamins and food minerals increased by 1.67 and 1.05 times, respectively, followed by food protein, food carbohydrate, food energy, and food fat production, which increased by 58.24%, 49.70%, 49.26%, and 48.70%, respectively. In food energy and food carbohydrate increase, cereal made the largest contribution, accounting for 41.98% and nearly 50%, respectively. In food protein production, cereal, vegetable, aquatic product, and livestock and poultry protein increases were the main sources of food protein increases (83.50%), accounting for 32.39%, 19.09%, 17.57%, and 14.45%, respectively. In food fat production, oil crop, livestock and poultry, and cereal fat increases were the main sources of food fat increases (80.86%), accounting for 37.35%, 29.33%, and 14.19%, respectively. In the increase of food vitamins, the increase of vegetable vitamins accounted for more than 60%. In the production of food minerals, the increase of vegetable minerals accounted for nearly 50% of the increase of food minerals.

During the study period, the changes of various food nutrient production structure showed significant differences. In food energy production, cereal was always the food group with the highest proportion of production, but the proportion decreased from 69.54% in 1995 to 60.45% in 2020. In food protein production, cereal was also always the food group with the highest proportion of production, but the proportion decreased from 54.47% in 1995 to 46.34% in 2020. In food carbohydrate production, the proportion of cereal carbohydrate production remained stable at about 80%, but the proportion decreased from 85.34% in 1995 to 73.52% in 2020. In food fat production, livestock and poultry was always the food group with the highest proportion of production, and the proportion was always close to 40%. In the production of food vitamins, vegetable was always the food group with the highest proportion of production, and the proportion increased from 55.13% in 1995 to 60.14% in 2020. Followed by cereal, the proportion decreased from 26.99% to 19.11%. In the production of food minerals, the food group with the highest proportion of production changed from cereal to vegetable. The proportion of the production of cereal minerals decreased from 41.85% in 1995 to 30.23% in 2020, while that of the production of vegetable minerals increased from 26.56% to 37.68%.

During the study period, the productions of various food nutrients have shown increasing trends in all regions. In food carbohydrate, food energy, and food protein production, the production in NEC increased by 1.20, 1.13, and 1.00 times, respectively. The growth rate of food carbohydrate, food energy, and food protein production in other regions ranged from 24.03% to 96.78%, from 27.08% to 91.20%, and from 36.91% to 81.69%, respectively. In food fat production, the production in NWC, NEC, SWC, NC, and CSC increased by 87.92%, 87.01%, 82.05%, 71.12%, and 52.84%, respectively, while production in EC increased by 3.98%. In the production of food vitamins, the production in NWC, SWC, CSC, EC, and NC increased by 3.31, 2.99, 2.09, 1.23, and 1.04 times, respectively, while production in NEC increased by 93.71%. In the production of food minerals, the production in NWC, SWC, and CSC increased by 1.82, 1.47, and 1.25 times, respectively, while production in NC, NEC, and EC increased by 91.14%, 84.87%, and 76.14%, respectively.

**Figure 4:**
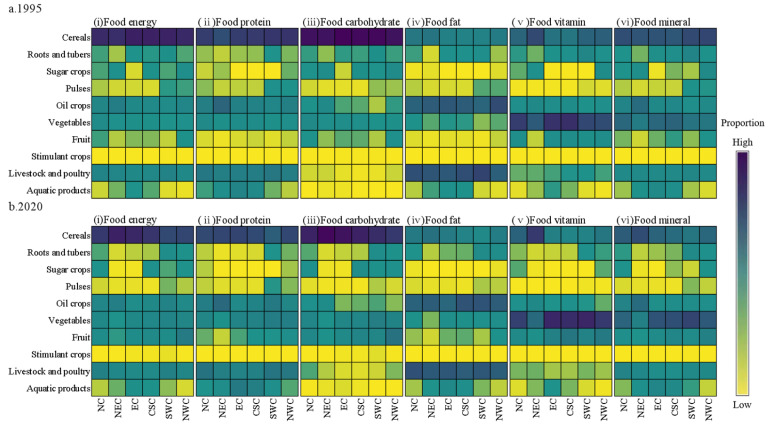
Food energy, food protein, food carbohydrate, food fat, food vitamin, and food mineral production structures of the six regions in (**a**) 1995 and (**b**) 2020.

At the regional scale, the changes of various food nutrient production structures also showed significant differences (Figure 4). In food energy, food protein, and food carbohydrate production, cereal was the food group with the highest proportion of production in all regions, accounting for more than 40%, 30%, and 50%, respectively. In food fat production, the largest source of NC and NEC was livestock and poultry fat, and the proportion was always close to 40%; the largest source of NWC was oil crop fat, but the proportion decreased from 45.70% in 1995 to 34.93% in 2020; in EC, the largest source changed from oil crop fat to livestock and poultry fat, and the proportion of oil crop fat decreased from 38.33% to 28.43%, while the proportion of livestock and poultry fat was always close to 40%; in CSC and SWC, the largest source changed from livestock and poultry fat to oil crop fat. The proportion of livestock and poultry fat decreased from 43.61% and 50.24% to 34.87% and 39.05%, while the proportion of oil crop fat increased from 35.11% and 29.76% to 41.63% and 40.77%, respectively. In the production of food vitamins, the largest source of NEC was cereal vitamins, and the proportion increased from 46.86% in 1995 to 56.48% in 2020; the largest source of other regions was always vegetable vitamins, and the proportions were all over 40%. In the production of food minerals, cereal was the food group with the highest proportion of production in NC and NEC, and the proportion of cereal minerals always accounted for nearly 40%; in EC, CSC, SWC, and NWC, the largest source changed from cereal minerals to vegetable minerals. The proportion of cereal minerals decreased from 40.99%, 38.98%, 47.03%, and 49.45% in 1995 to 31.17%, 26.20%, 19.19%, and 28.91% in 2020, while the proportion of vegetable minerals increased from 29.07%, 30.89%, 21.24%, and 19.42% to 40.68%, 43.33%, 49.60%, and 37.22%, respectively.

The production of various food nutrients produced in most provinces showed increasing trends. The production of food energy and food carbohydrate produced in 80% of the provinces showed increasing trends, and the production of Heilongjiang had the largest growth rate of 1.72 and 1.90 times, respectively. The production of food protein produced in nearly 90% of the provinces showed increasing trends, and the production of Inner Mongolia had the largest growth rate of 1.95 times. The production of food fat produced in more than 80% of the provinces showed increasing trends, and the production of Inner Mongolia had the largest growth rate of 2.09 times. The production of food vitamins produced in more than 90% of the provinces showed increasing trends, and the production of Guizhou had the largest growth rate of 4.17 times. The production of food minerals produced in 90% of the provinces showed increasing trends, and the production of Inner Mongolia had the largest growth rate of 2.53 times.

The changes of food nutrient production structure were also significantly different among the whole provinces. In food energy production, cereal energy was the largest source in more than 90% of the provinces, accounting for more than 50% in 1995 and more than 20% in 2020. In food protein production, cereal protein was the largest source in more than 70% of the provinces, accounting for more than 40% in 1995 and more than 20% in 2020. In food carbohydrate production, cereal carbohydrate was the largest source in all provinces, accounting for more than 70% and 30%, respectively, in 1995 and 2020. In food fat production, livestock and poultry fat was the largest source in nearly 60% of the provinces, accounting for more than 30% in 1995 and more than 20% in 2020. In the production of food vitamins, vegetable vitamins were the largest source in 90% of the provinces, accounting for more than 30% in 1995 and more than 40% in 2020. In the production of food minerals, the largest source of food minerals changed from cereal minerals to vegetable minerals in nearly 50% of the provinces, and cereal minerals and vegetable minerals accounted for more than 30% and 10%, respectively, in 1995, while that in 2020 accounted for more than 5% and 30%, respectively.

### 3.3. Characteristics of the Gravity Center Migration in Food Production

**Figure 5:**
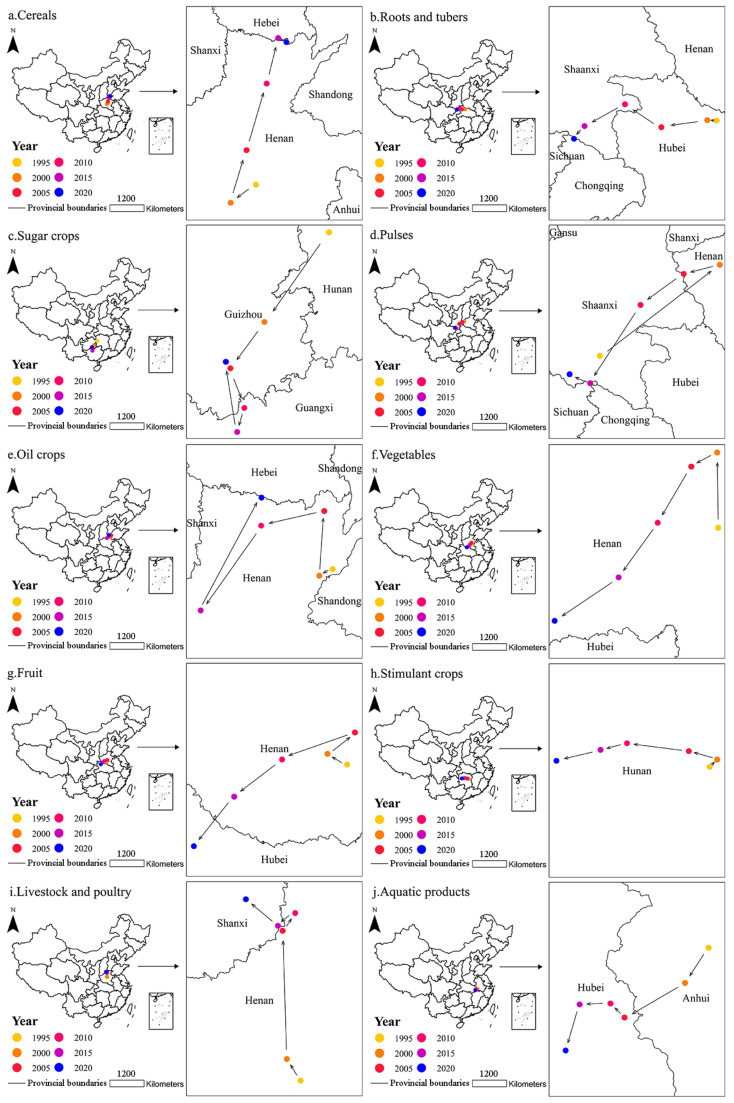
Migration tracks of gravity centers of (**a**) cereal, (**b**) root and tuber, (**c**) sugar crop, (**d**) pulse, (**e**) oil crop, (**f**) vegetable, (**g**) fruit, (**h**) stimulant crop, (**i**) livestock and poultry, and (**j**) aquatic product production in China during 1995–2020.

In this paper, the coordinates of the gravity centers of food production in China every five years as a time node during the study period and the migration tracks, movement angles, and distances of the gravity centers were obtained through the gravity center model (Figure 5, Table 3).

#### 3.3.1. Cereals

During 2000–2015, the gravity center of cereal production showed a significant characteristic moving northeast. From 1995 to 2000, the national cereal production showed a downward trend, but cereal production of Chongqing, Henan, and Yunnan increased by 0.18 × 10^8^ tons. Therefore, the gravity center of cereal production shifted 54 km from west to south, at an angle of 36°; from 2000 to 2005, the cereal production of Jilin, Henan, and Liaoning accounted for 34.80%, 24.48%, and 24.30% of the increase of national cereal production, respectively, so the gravity center of cereal production shifted 96 km from east to north, at an angle of 73°; from 2005 to 2010, the cereal production of Heilongjiang accounted for 31.05% in the increase of national cereal production, so the gravity center of cereal production shifted 122 km from east to north, at an angle of 73°; from 2010 to 2015, the ratio of cereal increment in Heilongjiang was still the highest, reaching 20.05%, followed by that in Jilin, accounting for 12.00%. Therefore, the gravity center of cereal production shifted 82 km from east to north, at an angle of 76°, moving from Henan to Hebei; from 2015 to 2020, the national cereal production showed a downward trend, but cereal production of Henan, Shandong, Liaoning, Guangdong, and Jiangsu increased by 0.07 × 10^8^ tons. The gravity center of cereal production shifted 16 km from east to south, at an angle of 27°, returning to Henan.

#### 3.3.2. Roots and Tubers

During the study period, the gravity center of root and tuber production tended to move westward. From 1995 to 2000, root and tuber production of Chongqing accounted for 56.64% of the increase in national root and tuber production. The gravity center of root and tuber production shifted 20 km from west to north, at an angle of 2°; from 2000 to 2005, national root and tuber production showed a downward trend, but root and tuber production of Gansu, Sichuan, Chongqing, and Yunnan increased by 0.02 × 10^8^ tons. The gravity center of root and tuber production shifted 97 km from west to south, at an angle of 9°; from 2005 to 2010, national root and tuber production showed a downward trend, but root and tuber production of Guizhou, Shaanxi, Inner Mongolia, Ningxia, Qinghai, and Xinjiang increased by 0.01 × 10^8^ tons. The gravity center of root and tuber production shifted 91 km from west to north, at an angle of 32°, moving from Hubei to Shaanxi; from 2010 to 2015, national root and tuber production showed a downward trend, but root and tuber production of Sichuan and Guizhou increased by 0.01 × 10^8^ tons, so the gravity center of root and tuber production shifted 96 km from west to south, at an angle of 28°; from 2015 to 2020, the root and tuber production of Hebei, Sichuan, Gansu, Shanxi, Yunnan, Guizhou, and Chongqing accounted for 96.42% in the increase of national root and tuber production. The gravity center of root and tuber production shifted 35 km from west to south, at an angle of 50°, moving from Shaanxi to Chongqing.

#### 3.3.3. Sugar Crops

The gravity center of sugar crop production tended to move southwest. From 1995 to 2000, the national sugar crop production showed a downward trend, but the sugar crop production of Guangxi, Yunnan, and Guizhou increased by 0.08 × 10^8^ tons. The gravity center of sugar crop production shifted 193 km from west to south, at an angle of 54°, moving from Hunan to Guizhou; from 2000 to 2005, the sugar crop production of Guangxi accounted for 122.03% of the increase of national sugar crop production, so the gravity center of sugar crop production shifted 100 km from west to south, at an angle of 54°; from 2005 to 2010, the sugar crop production of Guangxi accounted for 96.25% in the increase of national sugar crop production. The gravity center of sugar crop production shifted 74 km from east to south, at an angle of 71°, moving from Guizhou to Guangxi; from 2010 to 2015, the national sugar crop production showed a downward trend, but the sugar crop production in Guangxi increased 0.01 × 10^8^ tons, with the largest increase in over 30% of the provinces. The gravity center of sugar crop production shifted 44 km from west to south, at an angle of 73°; from 2015 to 2020, the sugar crop production of Inner Mongolia, Guangxi, and Xinjiang accounted for 52.60%, 41.85%, and 31.55% of the increase of national sugar crop production, respectively. The gravity center of sugar crop production shifted 125 km from west to north, at an angle of 81°, returning to Guizhou.

#### 3.3.4. Pulses

During 2000–2015, the gravity center of pulse production showed a significant characteristic moving southwest. From 1995 to 2000, the pulse production of Heilongjiang accounted for 94.34% of the increase in national pulse production, so the gravity center of pulse production shifted 316 km from east to north, at an angle of 37°, moving from Shaanxi to Henan; from 2000 to 2005, the pulse production of Yunnan and Sichuan accounted for 50.65% and 43.80% of the increase of national pulse production, respectively. The gravity center of pulse production shifted 78 km from west to south, at an angle of 14°; from 2005 to 2010, the national pulse production showed a downward trend, but the pulse production of Xinjiang and Hainan increased by 93,700 tons, so the gravity center of pulse production shifted 111 km from west to south, at an angle of 36°; from 2010 to 2015, the national pulse production showed a downward trend, but the pulse production in Yunnan increased 276,500 tons, with the largest increase in over 20% of the provinces. The gravity center of pulse production shifted 196 km from west to south, at an angle of 57°, moving from Shaanxi to Sichuan; from 2015 to 2020, the pulse production of Gansu accounted for the highest proportion of the increase of national pulse production, reaching 27.46%. The gravity center of pulse production shifted 47 km from west to north, at an angle of 24°, returning to Shaanxi.

#### 3.3.5. Oil Crops

The gravity center of oil crop production tended to move westward to some extent, and it was always located in Henan. From 1995 to 2000, the oil crop production of Anhui and Hubei accounted for 13.46% and 11.62% of the increase of national oil crop production, respectively. The gravity centers of oil crop production shifted 16 km from west to south, at an angle of 26°; from 2000 to 2005, the oil crop production of Heilongjiang accounted for 90.75% of the increase in national oil crop production, so the gravity centers of oil crop production shifted 68 km from east to north, at an angle of 86°; from 2005 to 2010, the national oil crop production showed a downward trend, but the oil crop production of Henan and Sichuan increased by 0.02 × 10^8^ tons, so the gravity centers of oil crop production shifted 68 km from west to south, at an angle of 13°; from 2010 to 2015, the national oil crop production showed a downward trend, but the production in nearly 40% of the provinces showed increasing trends. Among them, the oil crop production of Sichuan increased by 594,400 tons, so the gravity centers of oil crop production shifted 109 km from west to south, at an angle of 54°; from 2015 to 2020, the oil crop production of Heilongjiang accounted for 45.20% of the increase of national oil crop production, so the gravity centers of oil crop production shifted 134 km from east to north, at an angle of 62°.

#### 3.3.6. Vegetables

During 2000–2020, the gravity center of vegetable production had a significant trend moving southwest, and it was always located in Henan. From 1995 to 2000, the vegetable production of Shandong, Henan, Hebei, and Hubei accounted for 49.32% of the increase of national vegetable production, so the gravity center of vegetable production shifted 79 km from west to north, at an angle of 89°; from 2000 to 2005, the national vegetable production showed an increasing trend, but the vegetable production of Heilongjiang, Beijing, Shanxi, and Jilin decreased by 0.03 × 10^8^ tons, so the gravity center of vegetable production shifted 31 km from west to south, at an angle of 29°; from 2005 to 2010, the vegetable production of Henan, Jiangsu, Hunan, and Sichuan accounted for 108.18%, 77.37%, 63.47%, and 60.51% of the increase of national vegetable production, respectively. The gravity center of vegetable production shifted 69 km from west to south, at an angle of 59°; from 2010 to 2015, the vegetable production of Jiangsu, Yunnan, Sichuan, and Guangxi accounted for 41.96% of the increase of national vegetable production, so the gravity center of vegetable production shifted 70 km from west to south, at an angle of 54°; from 2015 to 2020, the vegetable production of Guizhou, Guangxi, and Sichuan accounted for 33.97% of the increase of national vegetable production, so the gravity center of vegetable production shifted 81 km from west to south, at an angle of 34°.

#### 3.3.7. Fruit

During 2005–2020, the gravity center of fruit production had a relatively significant trend moving southwest. From 1995 to 2000, the fruit production of Shandong, Hebei, Shaanxi, Henan, and Shanxi accounted for 54.52% of the increase of national fruit production, so the gravity center of fruit production shifted 31 km from west to north, at an angle of 29°; from 2000 to 2005, the fruit production of Shandong, Henan, and Hebei accounted for 36.44% of the increase of national fruit production, so the gravity center of fruit production shifted 49 km from east to north, at an angle of 38°; from 2005 to 2010, the fruit production of Shaanxi, Xinjiang, Henan, Guangxi, and Yunnan accounted for 49.31% of the increase of national fruit production, so the gravity center of fruit production shifted 109 km from west to south, at an angle of 20°; from 2010 to 2015, the proportion of the increase of fruit production in Guangxi was the highest, accounting for 12.12%, so the gravity center of fruit production shifted 85 km from west to south, at an angle of 38°; from 2015 to 2020, Guangxi still accounted for the highest proportion of the increase of national fruit production, reaching 28.62%. The gravity center of fruit production shifted 90 km from west to south, at an angle of 51°, moving from Henan to Hubei.

#### 3.3.8. Stimulant Crops

During 2000–2020, the gravity center of stimulant crop production had a significant trend moving westward, and it was always located in Hunan. From 1995 to 2000, the stimulant crop production of Fujian and Hubei accounted for 33.16% and 26.00% of the increase of national stimulant crop production, respectively. The gravity center of stimulant crop production shifted 15 km from east to north, at an angle of 45°; from 2000 to 2005, the stimulant crop production of Fujian, Sichuan, Yunnan, Zhejiang, and Hubei accounted for 74.75% of the increase in national stimulant crop production, so the gravity center of stimulant crop production shifted 41 km from west to north, at an angle of 16°; from 2005 to 2010, the stimulant crop production of Yunnan, Hubei, and Sichuan accounted for 46.27% of the increase of national stimulant crop production, so the gravity center of stimulant crop production shifted 87 km from west to north, at an angle of 7°; from 2010 to 2015, the proportion of the increase of stimulant crop production in Yunnan was the highest, accounting for 19.47%. The gravity center of stimulant crop production shifted 38 km from west to south, at an angle of 14°; from 2015 to 2020, the stimulant crop production of Fujian, Hubei, Sichuan, Yunnan, and Guizhou accounted for 75.24% of the increase in national stimulant crop production, so the gravity center of stimulant crop production shifted 64 km from west to south, at an angle of 14°.

#### 3.3.9. Livestock and Poultry

The gravity center of livestock and poultry production tended to move northward. From 1995 to 2000, the national livestock and poultry production showed an increasing trend, but the livestock and poultry production of Sichuan, Jiangxi, Hubei, Heilongjiang, and Gansu decreased by 0.01 × 10^8^ tons. The gravity center of livestock and poultry production shifted 27 km from west to north, at an angle of 58°; from 2000 to 2005, the livestock and poultry production of Inner Mongolia accounted for 17.93% of the increase in national livestock and poultry production, so the gravity center of livestock and poultry production shifted 134 km from west to north, at an angle of 88°; from 2005 to 2010, the livestock and poultry production of Inner Mongolia, Heilongjiang, Henan, and Liaoning accounted for 34.48%, 20.34%, 19.88%, and 19.85% of the increase of national livestock and poultry production, respectively. The gravity center of livestock and poultry production shifted 23 km from east to north, at an angle of 54°; from 2010 to 2015, the livestock and poultry production of Henan, Shandong, Hebei, Hubei, and Yunnan accounted for 52.55% of the increase of national livestock and poultry production. The gravity center of livestock and poultry production shifted 22 km from west to south, at an angle of 36°, moving from Henan to Shanxi; from 2015 to 2020, the national livestock and poultry production showed a downward trend, but the livestock and poultry production of Ningxia, Xinjiang, Shanxi, Yunnan, Gansu, Tibet, Qinghai, and Guizhou increased by 0.03 × 10^8^ tons, so the gravity center of livestock and poultry production shifted 44 km from west to north, at an angle of 40°.

#### 3.3.10. Aquatic Products

The gravity center of aquatic product production had a trend moving southwest. From 1995 to 2000, the aquatic product production of Shandong, Fujian, and Guangdong accounted for 46.91% of the increase in national aquatic product production, so the gravity center of aquatic product production shifted 24 km from west to south, at an angle of 56°; from 2000 to 2005, the aquatic product production of Guangdong and Hubei accounted for 13.67% and 12.15% of the increase of national aquatic product production, respectively. The gravity center of aquatic product production shifted 39 km from west to south, at an angle of 30°, moving from Anhui to Hubei; from 2005 to 2010, the national aquatic product production showed an increasing trend, but the production of Fujian, Shanghai, Zhejiang, and Guangxi decreased by 881,000 tons. The gravity center of aquatic product production shifted 11 km from west to north, at an angle of 45°; from 2010 to 2015, the aquatic product production of Shandong and Guangdong accounted for 11.15% and 10.38% of the increase of national aquatic product production, respectively. The gravity center of aquatic product production shifted 17 km from west to south, at an angle of 2°; from 2015 to 2020, the national aquatic product production showed a downward trend, but the production of Fujian, Sichuan, and Guangdong increased by 0.01 × 10^8^ tons. The gravity center of aquatic product production shifted 27 km from west to south, at an angle of 73°.

### 3.4. Characteristics of the Gravity Center Migration in Nutrient Production

**Figure 6:**
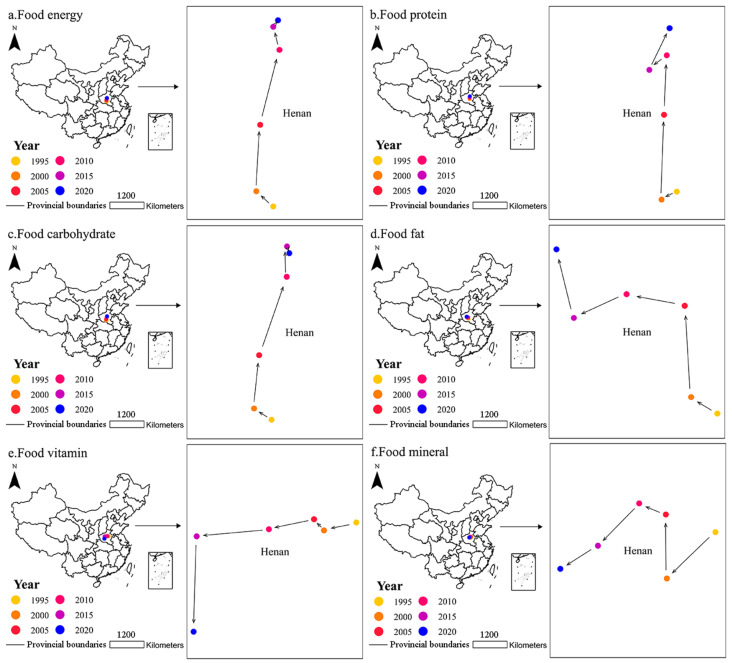
Migration tracks of gravity centers of (**a**) food energy, (**b**) food protein, (**c**) food carbohydrate, (**d**) food fat, (**e**) food vitamin, and (**f**) food mineral production in China during 1995–2020.

The migration tracks, angles, and distances of the gravity centers of nutrient production in China are as follows (Figure 6, Table 4). During the study period, the production of food energy of Henan and Heilongjiang increased by 2.61 × 10^14^ kcal, accounting for 32.31% of the increase in national total food energy production. The gravity center of food energy production shifted 16, 47, 54, 17, and 6 km from the west, east, east, west, and east to the north, at angles of 42°, 87°, 76°, 75°, and 54°, respectively.

The food protein production of Henan, Heilongjiang, and Inner Mongolia increased by 12.14 × 10^6^ tons, accounting for 35.43% of the increase in national total food protein production. The gravity center of food protein production shifted 9, 45, 31, 12, and 24 km from the west, east, east, west, and east to the south, north, north, south, and north, at angles of 27°, 88°, 87°, 40°, and 64°, respectively. The food carbohydrate production of Henan, Heilongjiang, Anhui, Shandong, Jiangsu, and Inner Mongolia increased by 8.93 × 10^7^ tons, accounting for 61.05% of the increase in national total food carbohydrate production. The gravity center of food carbohydrate production shifted 18, 47, 73, 27, and 6 km from the west, east, east, east, and east to the north, north, north, north, and south, at angles of 32°, 85°, 71°, 89°, and 71°, respectively. The food fat production of Henan, Heilongjiang, and Inner Mongolia increased by 5.40 × 10^6^ tons, accounting for 34.82% of the increase in national total food fat production. The gravity center of food fat production shifted 13, 38, 25, 24, and 30 km from the west to the north, north, north, south, and north, at angles of 32°, 86°, 11°, 24°, and 76°, respectively.

The food vitamin production of Henan, Shandong, Guangxi, Jiangsu, and Sichuan increased by 8.80 × 10^4^ tons, accounting for 37.98% of the increase in national total food vitamin production. The gravity center of food vitamin production shifted 29, 13, 40, 63, and 83 km from the west to the south, north, south, south, and south, at angles of 14°, 48°, 13°, 6°, and 88°, respectively. In food mineral production, the gravity center shifted 35, 33, 15, 31, and 23 km from the west to the south, north, north, south, and south, at angles of 43°, 89°, 23°, 46°, and 31°, respectively.

## 4. Discussion

Under the comprehensive implementation of various national policies and measures to promote the development of food production, as well as the improvement of the policy on cultivated land protection [40,41], ecological management optimization [42], expansion of effective irrigated areas [43,44], enhancements in agricultural production [45,46], livestock breeding [47], and aquaculture technology [48], etc., China’s food production have experienced exponential growth and a shift in production structure from cereal dominance to increased diversification. Production determines consumption, and, in turn, consumption also has a negative effect on production. It can be seen that this phenomenon is also the most direct reflection of the change in the consumption structure of Chinese residents.

Combined with the government’s financial project support, tax incentives, and technical guidance for regional food production in recent years, food production in the six regions has notably increased. China has a vast territory, and natural resources vary significantly due to climatic conditions and regional endowments in different regions [49,50]. Whether it is resource-intensive, labor-intensive, technology-intensive, or capital-intensive food production, natural resources will, to some extent, determine the regional comparative advantage and eventually promote the formation of different food production structures in different regions. For instance, East China, Central-South China, Southwest China, and Northwest China can leverage their comparative advantages, forming more distinctive food production structures.

Proactively adjusting production structure is one of the five fundamental tasks outlined in the Development of the Western Region [51], which has positively impacted food production increases in Xinjiang, Ningxia, Inner Mongolia, Shaanxi, Gansu, and Guizhou. As a representative agricultural area in China, Henan has continuously consolidated the foundation of agricultural development and formed an agricultural production mode characterized by high input and intensification [52], resulting in substantial growth in food production. Vegetables are a significant food group in China. The central government has issued various regulations and control policies on vegetable production, including policies on standardized production of “vegetable basket” products and ensuring vegetable supply, policies on strengthening quality and safety, policies on scientific and technological research, development and promotion, the establishment of a “standard park”, agricultural insurance, and other financial support policies, etc. These policies have successfully facilitated the development of the vegetable industry in most provinces. Additionally, cash crop cultivation has become a necessary means to address the “three rural problems” and develop the rural economy. Shaanxi, with its advantageous production conditions, has risen rapidly in the fruit industry in recent years. It has become one of the fastest-growing and most profitable industries in the rural economy, leading to significant changes in the food production structure in Shaanxi [53].

With the improvement of Chinese living standards, there has been increased attention toward nutrition, leading to a significant rise in nutrient production. Scholars have pointed out in their studies that income growth can result in increased intake of food protein and food fat, and it will improve the insufficient intake of food nutrients such as calcium and vitamins [54,55]. The unreasonable dietary structure is likely to lead to overweight, obesity, and chronic diseases, etc. [56,57]. For example, excessive energy intake leads to nutritional excess, while the inadequate intake of micronutrients, such as calcium, iron, and vitamins, leads to nutritional deficiency.

The overall northeastward movement of the gravity centers of cereal production basically aligns with the findings of Hou et al. [58]. Heilongjiang, as a major grain-producing province in China, shoulders the responsibility for stabilizing grain security. During the study period, Heilongjiang’s cereal production increased by 0.45 × 10^8^ tons, accounting for the highest proportion of the increase of China’s cereal production, reaching 22.74%. Zhao et al. emphasized that technological progress is the primary factor to improve grain production efficiency in Heilongjiang [59]. Moreover, in recent years, some forests, grasslands, and unused land have been transformed into cultivated land [60]. Even when China’s total cultivated land decreased from 1999 to 2008, the cultivated land area in Heilongjiang increased [61]. This expansion has indirectly influenced the positions of gravity centers of cereal production.

The gravity centers of vegetable production moving southwest is largely consistent with the conclusion obtained by Wang et al. [62]. From 1995 to 2020, national vegetable production exhibited an increasing trend, while the vegetable production of Beijing, Tianjin, and Jilin decreased by 0.05 × 10^8^ tons. In recent years, the western region has expanded its vegetable planting area through the development and utilization of land and water resources, as well as improvements in scientific and technological levels [63]. The southern region has also utilized significant amounts of land for cash crops like vegetables and fruit [21], thereby impacting the shift of the gravity center of vegetable and fruit production. Furthermore, factors such as dynamical investment [64], green supply chain management [65], and ecological environmental protection [66] will also have some impact on the relocation of the gravity center of food production.

Since nutrient production is derived from food, the migration trajectories of the gravity centers of food nutrient production are somewhat similar to those of food production. For example, cereal energy, cereal protein, and cereal carbohydrate production accounted for more than 50%, 40%, and 70% of food energy, food protein, and food carbohydrate production, respectively. Therefore, the migration trajectories of the gravity centers of food energy, food protein, and food carbohydrate production are similar to the movement of cereal production toward the north. Oil crop fat and livestock and poultry fat production contributed to over 30% of food fat production, causing the gravity centers of food fat production to move northwest. Vegetable vitamins production consistently represented over half of the food vitamin production, leading to a southwestward movement of the gravity center of food vitamin production.

This study incorporated 43 food items across 10 food groups and fully considered the macronutrients and micronutrients hidden in food, offering insights into the spatiotemporal pattern evolution of food and nutrient production in China. Nevertheless, this paper failed to consider the inter-provincial trade, supply–demand balance, and future development of food and nutrient production in China. Future research should comprehensively consider the systemic issues of food safety, conduct a supply–demand analysis, and forecast the future development of food and nutrient production in China. Additionally, future studies should also consider the production of cereals and oil crops, vegetables and fruit, livestock and poultry, and aquatic products, respectively, and fully consider the driving mechanism of spatiotemporal changes in food and nutrition production with a quantitative method.

## 5. Conclusions

This paper has uncovered the spatiotemporal pattern evolution of food and nutrient production in China from 1995 to 2020, based on the conversion of food nutrients and gravity center models for cereal, root and tuber, sugar crop, pulse, oil crop, vegetable, fruit, stimulant crop, livestock and poultry, and aquatic products, as well as food energy, food protein, food carbohydrate, food fat, food vitamins, and food mineral production. The significant increase in food production can be attributed to national policies, the optimization of production conditions, and technological progress. Profiting from an integrated effect of natural resources, policies, and economy, China’s food production structure has become more balanced, with noticeable changes observed in the food production structure of EC, CSC, SWC, NWC, and the majority of provinces. National food vitamins, food minerals, food protein, food carbohydrate, food energy, and food fat production increased by 1.67 times, 1.05 times, 58.24%, 49.70%, 49.26%, and 48.70%, respectively. Increasing trends in all types of food nutrient production were observed in the six regions and most provinces. Moreover, the food fat production structure in EC, CSC, and SWC had significant changes; the food mineral production structure in China, EC, CSC, SWC, NWC, and approximately 50% of the provinces experienced substantial changes. Notably, the food group with the highest proportion of production shifted from cereal to vegetable.

Under the comprehensive influence of ever-changing natural, political, social, economic, and technological factors, the gravity centers of food and nutrient production in China have continuously shifted in different directions. Overall, the gravity center of cereal production moved northeast, which may pose increased pressure on water resources and the ecological environment in water-scarce northern regions. Additionally, traditional food production areas have gradually lost their advantages due to factors such as sowing area, economic level, and agricultural production technology. Therefore, relevant departments should introduce policies and measures to revitalize and develop traditional food production areas, including infrastructure improvements. In addition, it is imperative to adjust the distribution of various types of food production according to the different natural resource conditions in each region, thoroughly explore the optimal food production mode for each region and province, and rationally allocate the planting ratios of grain crops and cash crops to accurately enhance regional economic development. In comparison to the gravity centers of food production, the movement of gravity centers of food nutrient production was relatively limited and consistently located in Henan. To sum up, China’s food and nutrient production is gradually adapting to the ever-increasing social demands, with significant production increases and an optimized production structure. However, pertinent policies to promote the rationalization of food production are still urgently needed.

## Figures and Tables

**Table 1:** Food groups and food items of food production in China.

Food Group	Food Item
cereals	rice, wheat, maize, millet, sorghum, barley, other cereals
roots and tubers	potato, other roots and tubers
sugar crops	sugarcane, sugar beet
pulses	mung bean, adzuki bean, other pulses
oil crops	soybean, peanut, rapeseed, sunflower seed, flax seed, sesame, other oil crops
vegetables	nil
fruit	banana, apple, orange, pear, grape, pineapple, date, persimmon, watermelon, melon, strawberry, other fruit
stimulant crops	tea
livestock and poultry	pork, beef, mutton, milk, egg, honey
aquatic products	fishes, shrimps and crabs, shellfishes

**Table 2:** Six regions and 31 provinces (municipalities and autonomous regions, excluding Hong Kong, Macao, and Taiwan) in China.

Region	Province *
North China (NC)	Beijing, Tianjin, Hebei, Shanxi, Inner Mongolia
Northeast China (NEC)	Liaoning, Jilin, Heilongjiang
East China (EC)	Shanghai, Jiangsu, Zhejiang, Anhui, Fujian, Jiangxi, Shandong
Central-South China (CSC)	Henan, Hubei, Hunan, Guangdong, Guangxi, Hainan
Southwest China (SWC)	Chongqing, Sichuan, Guizhou, Yunnan, Tibet
Northwest China (NWC)	Shaanxi, Gansu, Qinghai, Ningxia, Xinjiang

* ‘Province’ includes the municipalities and autonomous regions, excluding Hong Kong, Macao, and Taiwan.

**Table 3:** Changes of the gravity centers of food production in China during 1995–2020.

Group	1995–2000		2000–2005		2005–2010		2010–2015		2015–2020	
	α * (°)	D * (km)	α * (°)	D * (km)	α * (°)	D * (km)	α * (°)	D* (km)	α * (°)	D * (km)
Cereals	36	54	73	96	73	122	76	82	−27	16
Roots and tubers	−2	20	9	97	−32	91	28	96	50	35
Sugar crops	54	193	54	100	−71	74	73	44	−81	125
Pulses	37	316	14	78	36	111	57	196	−24	47
Oil crops	26	16	86	68	13	68	54	109	62	134
Vegetables	−89	79	29	31	59	69	54	70	34	81
Fruit	−29	31	38	49	20	109	38	85	51	90
Stimulant crops	45	15	−16	41	−7	87	14	38	14	64
Livestock and poultry	−58	27	−88	134	54	23	36	22	−40	44
Aquatic products	56	24	30	39	−45	11	2	17	73	27

* ‘α’ means shift angle; ‘D’ means moving distance.

**Table 4:** Changes of the gravity centers of food nutrient production in China during 1995–2020.

Group	1995–2000		2000–2005		2005–2010		2010–2015		2015–2020	
	α * (°)	D * (km)	α * (°)	D * (km)	α * (°)	D * (km)	α * (°)	D * (km)	α * (°)	D * (km)
Food calorie	−42	16	87	47	76	54	−75	17	54	6
Food protein	27	9	88	45	87	31	40	12	64	24
Food carbohydrate	−32	18	85	47	71	73	89	27	−71	6
Food fat	−32	13	−86	38	−11	25	24	24	−76	30
Food vitamin	14	29	−48	13	13	40	6	63	88	83
Food mineral	43	35	−89	33	−23	15	46	31	31	23

* ‘α’ means shift angle; ‘D’ means moving distance.

## Data Availability

The datasets generated for this study are available on request to the corresponding author.
